# PPE26 induces TLR2-dependent activation of macrophages and drives Th1-type T-cell immunity by triggering the cross-talk of multiple pathways involved in the host response

**DOI:** 10.18632/oncotarget.5956

**Published:** 2015-10-02

**Authors:** Haibo Su, Cong Kong, Lin Zhu, Qi Huang, Liulin Luo, Honghai Wang, Ying Xu

**Affiliations:** ^1^ State Key Laboratory of Genetic Engineering, Institute of Genetics, School of Life Science, Fudan University, Shanghai, China; ^2^ Department of Clinical Laboratory Medicine, Shanghai Pulmonary Hospital, Tongji University School of Medicine, Shanghai, China

**Keywords:** PPE26, PE/PPE, mycobacterium tuberculosis, TLR2, proteomics, Immunology and Microbiology Section, Immune response and Immunity

## Abstract

The pathophysiological functions and the underlying molecular basis of PE /PPE proteins of *M. tuberculosis* remain largely unknown. In this study, we focused on the link between PPE26 and host response. We demonstrated that PPE26 can induce extensive inflammatory responses in macrophages through triggering the cross-talk of multiple pathways involved in the host response, as revealed by iTRAQ-based subcellular quantitative proteomics. We observed that PPE26 is able to specifically bind to TLR2 leading to the subsequent activation of MAPKs and NF-κB signaling. PPE26 functionally stimulates macrophage activation by augmenting pro-inflammatory cytokine production (TNF-α, IL-6 and IL-12 p40) and the expression of cell surface markers (CD80, CD86, MHC class I and II). We observed that PPE26-treated macrophages effectively polarizes naïve CD4^+^ T cells to up-regulate CXCR3 expression, and to secrete IFN-γ and IL-2, indicating PPE26 contributes to the Th1 polarization during the immune response. Importantly, rBCG::PPE26 induces stronger antigen-specific TNF-α and IFN-γ activity, and higher levels of the Th1 cytokines TNF-α and IFN-γ comparable to BCG. Moreover, PPE26 effectively induces the reciprocal expansion of effector/memory CD4^+^/CD8^+^ CD44^high^CD62L^low^ T cells in the spleens of mice immunized with this strain. These results suggest that PPE26 may be a TLR2 agonist that stimulates innate immunity and adaptive immunity, indicating that PPE26 is a potential antigen for the rational design of an efficient vaccine against *M. tuberculosis*.

## INTRODUCTION

*Mycobacterium tuberculosis* is considered to be one of the most successful and widespread intracellular pathogens, with approximately one-third of the world's population infected and 1.5 million deaths annually [1, 2]. Almost a century after its introduction, Bacille Calmette-Guérin (BCG) is still the only current widely used vaccine for protection against TB [3]. However, the major drawback of BCG is that it only protects against several forms of TB in toddlers and works erratically to protect adults and adolescents from pulmonary disease [4]. The failure of efforts to improve BCG lies in the fact that we do not understand what constitutes a protective immune response to TB [1, 5]. Recently, targeting the antigens of *Mycobacterium tuberculosis* has been considered to be crucial for both control of and protective immune response against the pathogen, and this may substantially improve the situation [6]. Consequently, to characterize *M. tuberculosis* antigen is reasonably essential to understand the connection between the host and the pathogen, and also can facilitate the development of prospective vaccines candidates [7–9].

Macrophages in the alveoli are thought to serve as the main effector cells during the early stages of infection with *M. tuberculosis*. Once the pathogen enters the lung via small aerosolized droplets exhaled by infected individuals, macrophages phagocytose the bacilli, transport it into deeper tissues and limit bacterial survival and proliferation [10]. However, *M. tuberculosis* can use multiple and even overlapping strategies to hide and replicate within permissive macrophages recruited to the lung. These strategies include blockage of phagocytosis, attenuation of macrophage antigen presentation, interference with cellular trafﬁcking and immune recognition, and manipulation of autophagy [11–15].

TLR2 on APCs initiates innate immune responses and modulates adaptive immune responses through the recognition of microbial molecules, which restricts *M. tuberculosis* replication and eventually leads to its elimination. TLR2 is reported to be recognized by *M. tuberculosis* components such as lipoproteins [16–21], peptidoglycan [22–24] and PE/PPE proteins [25–30]. Rv1818c was shown to interact directly with TLR2, thereby increasing the Th1 cytokine production [31]. Lipomannan (LM) from several mycobacterial species was found to activate macrophage characterized by TNF-α and nitric oxide secretion through TLR2 [32]. The binding of *M. tuberculosis* antigens to TLR2 through Toll/IL-1R homology domains results in recruitment of the adaptor molecules MyD88 and/or TRIF, ultimately leading to the activation of MAPKs and transcription factors (i.e., NF-κB and IRFs) [21, 33–35]. TLR2-dependent activation of macrophages/DC can up-regulate the expression of surface molecules (i.e., CD80, CD86, MHC I and MHC II), and induce the secretion of pro-inflammatory cytokines (i.e., TNF-α, IL-6 and IL-12) [36, 37]. Together, TLR2 engagement on APC or T cells can drive Th1 polarization and enhance effector functions or protective responses against *M. tuberculosis* [8, 30, 38].

PE (Pro-Glu) and PPE (Pro-Pro-Glu) are two gene families that account for almost 10% of the *M. tuberculosis* genome and include more than 160 members in *M. tuberculosis* [39]. PE and PPE family proteins are named for the presence of multiple repeats at their N-terminal domain, which are particularly critical for generating antigenic variation and evading host immune responses [40]. PE/PPE proteins have been linked to the induction of pro- or anti-inflammatory response by modulating the activation of macrophages. PPE18 was observed to induce anti-inflammatory response to suppress macrophage innate-effector functions [27]. PPE57 could trigger pro-inflammatory programming and drive Th1-type cytokine secretion in macrophages [29]. To determine the effects of PE/PPE proteins on macrophages gives insights into the pathogenesis associated with TB and may provide new strategies for protection against *M. tuberculosis.*

PPE26 (Rv1789) is one of the ESX-5-encoded PE/PPE proteins and has been found in the membrane fraction of *M. tuberculosis* [41]. PPE26 is a non-essential gene for the in vitro growth of H37Rv [42] and caused no significant difference in the growth rate of the rBCG::PPE26 strain compared with the BCG strain (Supplementary Figure S1B). Although comparative analyses suggest that PPE26 may be associated with pathogenesis [43, 44], the immunological function of this protein is not fully understood, especially with respect to its role in innate and adaptive immunity. Here, we attempted to clarify the precise mechanism by which PPE26 triggered the Th1-type immune response via macrophages activation. The iTRAQ-based subcellular quantitative proteomic changes and concurrent biological validations revealed that PPE26 induced macrophage activation by triggering TLR2-dependent cross-talk of multiple pathways involved in the host response. PPE26 could up-regulate macrophages function and induce the Th1 immune response. Moreover, immunization with rBCG::PPE26 effectively polarized T cells towards Th1 phenotype and promoted the proliferation of effector/memory CD4^+^/CD8^+^CD44^high^CD62L^low^ T cells. Deep comprehension of the PPE26 immunological functions in the host immune response may be useful for understanding host-pathogen interactions and the development of more effective vaccines.

## RESULTS

### PPE26 triggered the cross-talk among multiple signaling pathways downstream of TLR2, as revealed by iTRAQ-based subcellular quantitative proteomic

We designed an iTRAQ-based subcellular quantitative proteomic approach to identify proteins associated with PPE26 functions in macrophages (Supplementary Figure S2). As a result, 352 up-regulated or 214 down-regulated were found in the cytoplasmic fraction. Meanwhile, 281 up-regulated or 205 down-regulated were detected in the nuclear fraction (Supplementary Table 2-5, Supplementary Figure S3). The proteins that were differentially expressed in the cytosol and nucleus were associated with signal transduction, immunology and defense, response to tress and apoptosis (Supplementary Figure S4 and Figure S5). It is inferred that the PPE26 could trigger extensive inflammatory response in macrophages by eliciting a series of intracellular signaling cascades such as TLR signaling and NF-κB-regulated signaling.

**Figure 1:**
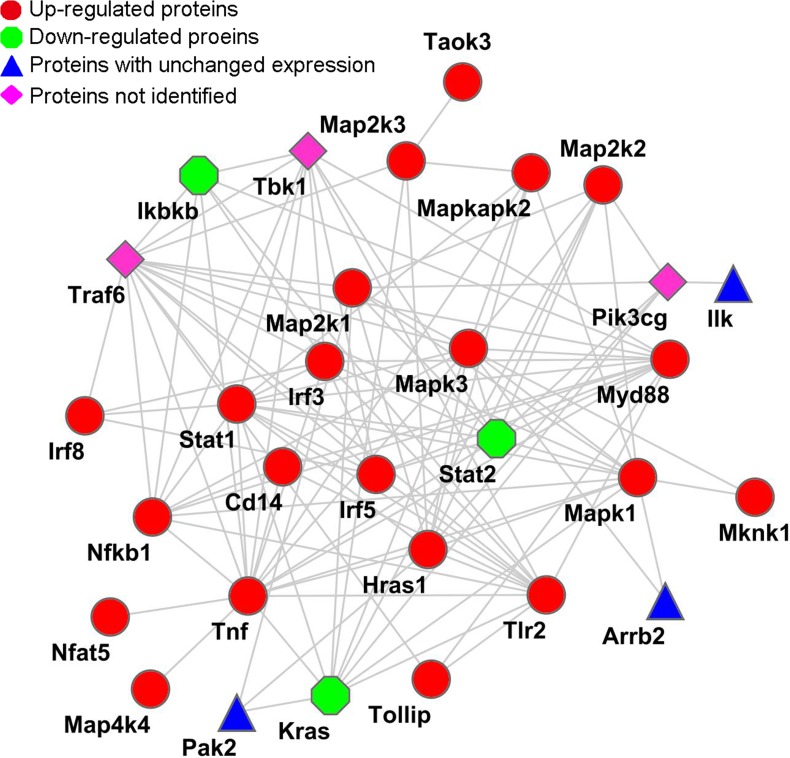
Reconstructed networks involving the NF-κB, MAPK, IRF, and TLR signaling pathway responses to PPE26 stimulation The key components (Table 1), such as NF-κB, MAPK, IRF, and TLR2, were acquired by PANTHER (http://www.pantherdb.org/) and submitted to STRING (http://string.embl.de/) for network construction. The network was modified using the Cytoscape 3.1.0 software.

To clarify the mechanism how PPE26 interacts with macrophages. PANTHER was used to determine the differentially expressed proteins engaged in the TLR2, MAPKs, NF-κB, and IRF signaling pathways (Table 1). Subsequently, STRING was used to reconstruct an interaction network. As shown in Figure 1. A possible cross-talk connection involving the key components of MAPKs, NF-κB, and IRF signaling may coordinate the modulation of inflammatory factors for the PPE26-induced TLR2-mediated early response.

**Table 1:** Quantified Proteins Involved in TLR2, NF-κB, MAPK and IRF Signaling Pathway

Uniprot-ID	Location^a^	Gene Symbol	Protein Discription	H/L Ratio^b^	Coveragege (95%)	Unique Peptides	*P* value
Q920X9	cytosol	CD14	CD14 antigen	3.83	15.16	5	0.0817
P23611	cytosol	Irf8	Interferon regulatory factor 8	2.44	6.93	8	0.0002
Q9WV30	cytosol	Nfat5	Nuclear factor of activated T-cells 5	4.01	5.52	9	0.0263
Q9WV30	nuclear	Nfat5	Nuclear factor of activated T-cells 5	2.95	27.13	2	0.0123
Q3U169	cytosol	Irf5	Interferon regulatory factor 5	2.83	9.07	11	0.0021
P63085	cytosol	Mapk1	Mitogen-activated protein kinase 1	1.58	47.21	5	0.0679
P63085	nuclear	Mapk1	Mitogen-activated protein kinase 1	1.81	12.09	2	0.0308
O08605	cytosol	Mknk1	MAP kinase-interacting serine/threonine-protein kinase 1	1.29	17.85	2	0.0688
O88351	cytosol	Ikbkb	Inhibitor of nuclear factor kappa-B kinase subunit beta	0.67	11.76	12	0.0522
P49138	cytosol	Mapkapk2	MAP kinase-activated protein kinase 2	1.24	5.84	4	0.0426
O09110	cytosol	Map2k3	Dual specificity mitogen-activated protein kinase kinase 3	1.78	30.43	1	0.0571
O09110	nuclear	Map2k3	Dual specificity mitogen-activated protein kinase kinase 3	1.35	7.14	1	0.0511
Q63844	cytosol	Mapk3	Mitogen-activated protein kinase 3	1.49	16.22	2	0.0169
Q3TW11	cytosol	Stat1	Signal transducer and activator of transcription	1.36	22.91	1	0.0451
Q3TW11	nuclear	Stat1	Signal transducer and activator of transcription	2.82	9.83	1	0.0147
P25799	cytosol	Nfkb1	Nuclear factor NF-kappa-B p105 subunit	1.61	44.48	3	0.0203
P25799	nuclaer	Nfkb1	Nuclear factor NF-kappa-B p105 subunit	2.55	12.77	1	0.0011
Q3UK05	cytosol	Map2k1	Mitogen activated protein kinase kinase 1	1.95	9.45	3	0.0058
P70671	cytosol	Irf3	Interferon regulatory factor 3	1.26	29.21	3	0.0118
P70671	nuclear	Irf3	Interferon regulatory factor 3	2.73	15.12	2	0.0726
Q63932	cytosol	Map2k2	Dual specificity mitogen-activated protein kinase kinase 2	1.72	32.24	4	0.0232
P97820	cytosol	Map4k4	Mitogen-activated protein kinase kinase kinase kinase 4	1.26	29.26	3	0.0118
Q811T5	cytosol	Tlr2	Toll-like receptor 2	1.54	13.78	7	0.0132
Q9WVL2	cytosol	Stat2	Signal transducer and activator of transcription 2	0.65	24.84	11	0.1102
P22366	cytosol	Myd88	Myeloid differentiation primary response protein MyD88	2.88	34.63	8	0.0234
Q3U593	cytosol	Tnf	Tumor necrosis factor	5.15	21.27	25	0.0367
Q8BYC6	cytosol	Taok3	Serine/threonine-protein kinase TAO3	2.53	11.91	6	0.0153
Q8CIN4	cytosol	Pak2	Serine/threonine-protein kinase PAK 2	1.12	3.29	2	0.1001
Q8C5G6	cytosol	Tollip	Toll-interacting protein	1.23	17.75	7	0.0552
Q61411	cytosol	Hras	GTPase HRas	1.92	14.58	13	0.0374
Q91YI4	cytosol	Arrb2	Beta-arrestin-2	1.07	8.78	5	0.0699
Q5J7N1	cytosol	Kras	Kras protein	0.76	2.59	15	0.0069

a Proteins identified in cytosol or nuclear fraction.

b Proteins expression changes of PPE26-stimulated (H) vs CONTROL(L) macrophages.

**Figure 2:**
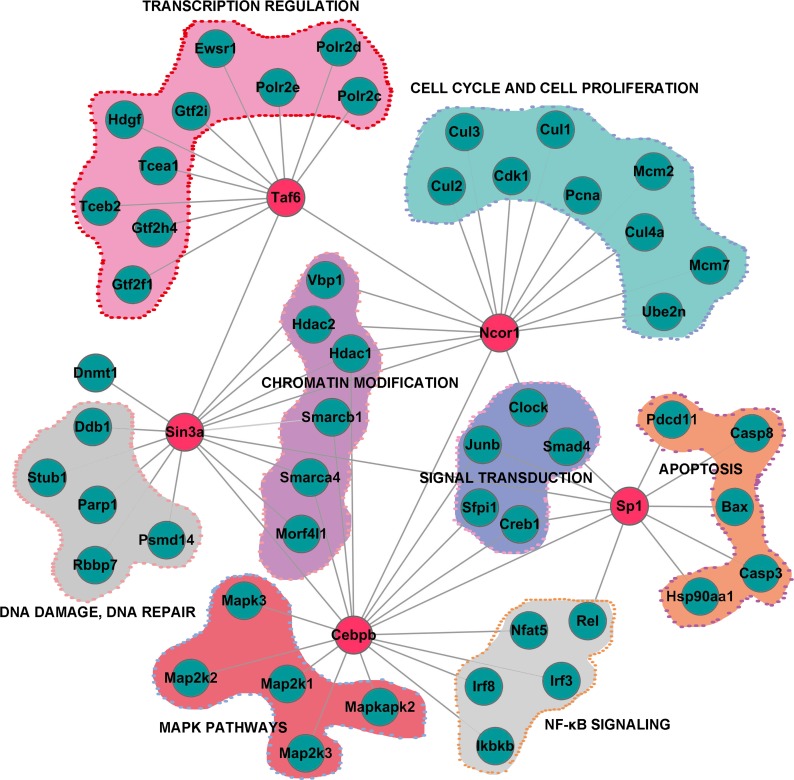
Subnetwork constructed by data-dependent network analysis of proteins involved in different biological processes The previously known functions of the proteins in the network were based on PANTHER (http://www.pantherdb.org/). The “zoom-in” map of the subnetwork was extracted from the global regulatory network involving Taf6, Sin3a, Ncor1, C/EBP-b, and Sp1 (Supplementary Figure S6).

The 120 transcription factors or cofactors (Supplementary Table 6) indentified in the nuclear fraction were mainly involved in transcription, immune system processes, signaling transduction and response to interferon-γ (Supplementary Figure S6). The global functional maps generated from data-dependent bioinformatics analysis illustrated how the TFs regulatory network operated in conjunction with upstream signal cascades to generate the response following PPE26 stimulation (Figure 2 and Supplementary Figure S7). Together, we hypothesized that PPE26 could bind to the cell surface receptor TLR2, thereby activating MAPKs, NF-κB and IRFs pathways (Supplementary Figure S8), and finally initiating pro-inflammatory programming in macrophages.

### PPE26 induces pro-inflammatory cytokine production through TLR2

**Figure 3:**
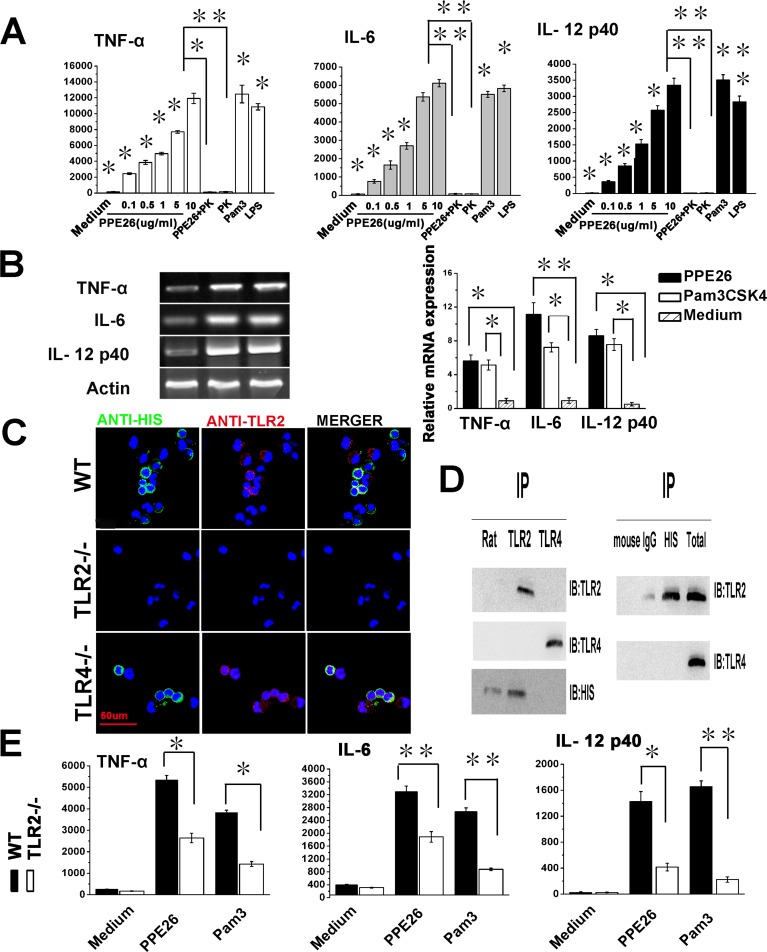
PPE26 induces cytokine production by mouse macrophages through TLR2 **A.**. RAW264.7 cells were incubated with various concentrations of PPE26 (0.1-10μg/ml), proteinase K (PK; 50 mg/ml), PPE26 (10μg/ml) + PK (50 mg/ml), Pam_3_CSK4 (5 mg/ml) (Pam3 = Pam_3_CSK4) or LPS (1μg/ml). After 24 h of incubation, supernatants were collected, and TNF-a, IL-6, and IL-12p40 levels were measured by ELISA. **B.**. Semiquantitative and quantitative RT-PCR analysis of mRNA levels for TNF-a, IL-6, and IL-12p40 in total RNA that was extracted from RAW264.7 cells incubated in medium alone, Pam_3_CSK4 (5 mg/ml) or PPE26 (10μg/ml). The mRNA levels were normalized to the β-actin mRNA level. **C.**. Macrophages derived from WT, TLR2^−/−^, and TLR4^−/−^ mice were incubated with PPE26-His (10μg/ml) for 1 h. After washing and staining, the cells were fixed and photographed by confocal microscopy. Scale bar, 50μm. **D.**. Macrophages derived from WT, TLR2^−/−^, and TLR4^−/−^ mice were treated with PPE26 (10μg/ml) for 6 h, cell lysates were immunoprecipitated with anti-rat IgG, anti-mouse IgG, anti-His, anti-TLR2, or anti-TLR4; then, proteins were visualized by immunoblotting with the anti-His, anti-TLR2, or anti-TLR4 Abs. Total cell lysate was used as an input control. **E.**. Macrophages derived from TLR2^−/−^ and WT mice were treated with medium, PPE26 (10μg/ml) or Pam_3_CSK4 (5 mg/ml). TNF-α, IL-6, and IL-12p40 levels were measured by ELISA. All data are expressed as the mean± SD from three separate experiments (*P < 0.05 or **P < 0.01).

To validate whether PPE26 induced macrophage activation and promoted pro-inflammatory cytokine production in macrophages, we measured cytokine levels in the culture supernatants of RAW264.7 cells treated with PPE26, LPS, Pam_3_CSK4 or proteinase K for 24 h by ELISA. As shown in Figure 3A, PPE26 significantly increased the production of TNF-α, IL-6, and IL-12 p40 in a dose-dependent manner. PPE26 also produced a similar increase in the relative expression of TNF-α, IL-6, and IL-12 p40 mRNA (Figure 3B). In contrast, proteinase K treatment abolished the PPE26-induced increase in TNF-α, IL-6, and IL-12 p40 production, indicating that PPE26 specifically induced macrophages to secrete cytokines.

In MS data, TLR2 were increased by 54% in macrophages infected with rBCG::PPE26 compared with the control group, indicating that TLR2 might be the interacting partner of PPE26. To clarify this, we examined whether PPE26 interacted with TLR2 using confocal microscopy. As shown in Figure 3C, PPE26 was observed to preferentially bind to TLR2 but not TLR4. Additionally, immunoprecipitation further confirmed this observation (Figure 3D). Next, we measured TNF-α, IL-6, and IL-12 p40 levels in the supernatants of PPE26-treated macrophages from WT and TLR2^−/−^ mice. We demonstrated that PPE26-induced cytokine production was significantly decreased in macrophages from TLR2^−/−^ mice compared with WT mice (Figure 3E). Our results suggested that PPE26 can stimulate macrophage activation and induce the production of pro-inflammatory cytokines through TLR2.

### PPE26-induced cytokines production is involved in the activation of MAPKs pathway

**Figure 4:**
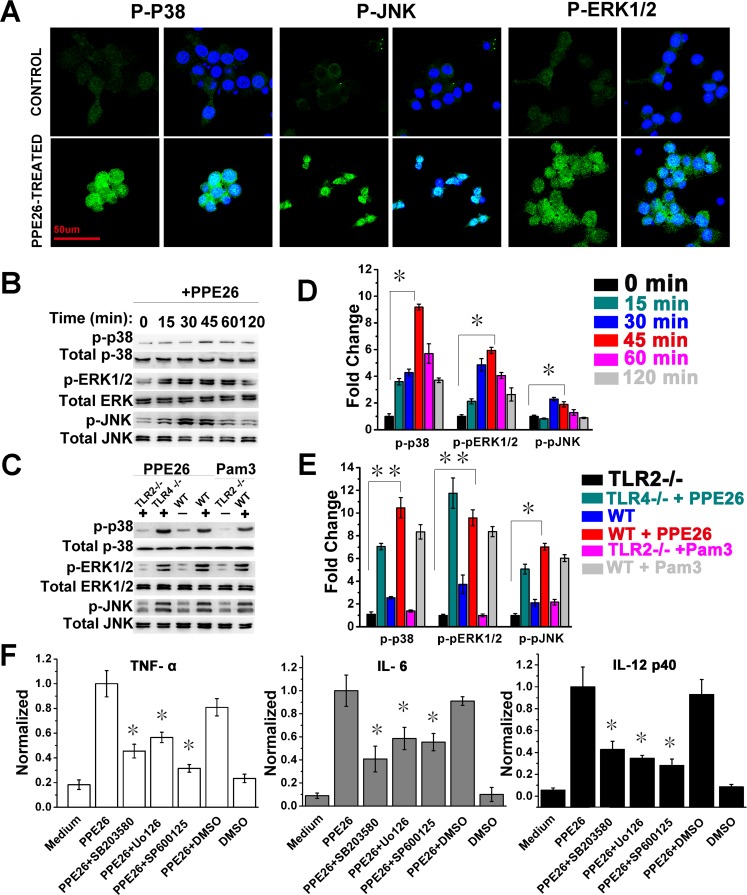
Macrophages activation triggered by PPE26 involves activation of MAPKs signaling **A.**. RAW264.7 cells were treated with PPE26 (10μg/ml) for 1 h. After washing and staining, the cells were fixed and photographed by confocal microscopy. Scale bar, 50μm. **B.** and **C.**. RAW264.7 cells were treated with PPE26 (10μg/ml) for the indicated time (0-120 min). Macrophages isolated from WT, TLR2^−/−^, or TLR4^−/−^ mice were treated with PPE26 (10μg/ml) for 1h. The phosphorylation of p38, ERK (1/2) and JNK were examined by blotting with specific antibodies to p-p38, p38, p-ERK1/2, ERK1/2, p-JNK and JNK. **D.** and **E.**. Densitometric analysis of the Western blot in B and C, respectively. Unstimulated cells were given a value 1.00. **F.**. RAW264.7 cells were treated with inhibitors of DMSO (vehicle control), p38 (SB203580, 10μM), ERK (U0126, 10μM) or JNK (SP600125, 10μM) for 1 h at 37°C, followed by incubation with PPE26 (10μg/ml) for 36 h. The amounts of TNF-a, IL-6, and IL-12p40 levels were measured by ELISA. All data are expressed as the mean± SD from three separate experiments (*P < 0.05 or **P < 0.01).

In our MS data, we found that Mitogen-activated protein kinase kinase 1 (MAP2K1), which can phosphorylate and activate ERK1 and ERK2, was increased by 95% in the cytosol. MAP kinase kinase kinase kinase 4 (MAP4K4) acts as a link to the JNK and was increased by 26% in the cytosol. We also found that MAP kinase kinase 3 (MAP2K3), an upstream kinase related to p38 activation, was increased by 78% in the cytosol and 35% in the nucleus. The evidence indicated that the ERK, JNK, and p38 cascades were activated following PPE26 stimulation. To clarify this, we examined the effect of PPE26 on MAPK activation by confocal microscopy and western blotting analysis. As shown in Figure 4A, PPE26 produced strong phosphorylation of p38, JNK, and ERK1/2. Next, western blot analysis also showed that the levels of the phosphorylation of MAPKs were obviously up-regulated, and the peak phosphorylation occurred within 45 min following PPE26 stimulation (Figure 4B and 4D). Moreover, PPE26-induced phosphorylation of p38, JNK and ERK1/2 were significantly attenuated in macrophages from WT and TLR4^−/−^ mice compared to macrophages from TLR2^−/−^ mice (Figure 4C and 4E). Thus, the phosphorylation of MAPKs in response to PPE26 is mediated primarily by TLR2, not by TLR4.

To determine the functional roles of MAPKs signaling in the context of PPE26-induced pro-inflammatory cytokine production, RAW264.7 cells were pretreated with a p38 inhibitor (SB203580), an ERK1/2 inhibitor (U0126), or a JNK inhibitor (SP600125) for 1 h prior to stimulation with PPE26. Levels of TNF-α, IL-6, and IL-12 p40 were measured by ELISA. We found that pharmacological inhibition of MAPKs significantly abrogated the PPE26-induced production of TNF-α, IL-6, and IL-12 p40 (Figure 4F).

### PPE26 induces the translocation of NF-κB subunits to the nucleus in mouse macrophages

**Figure 5:**
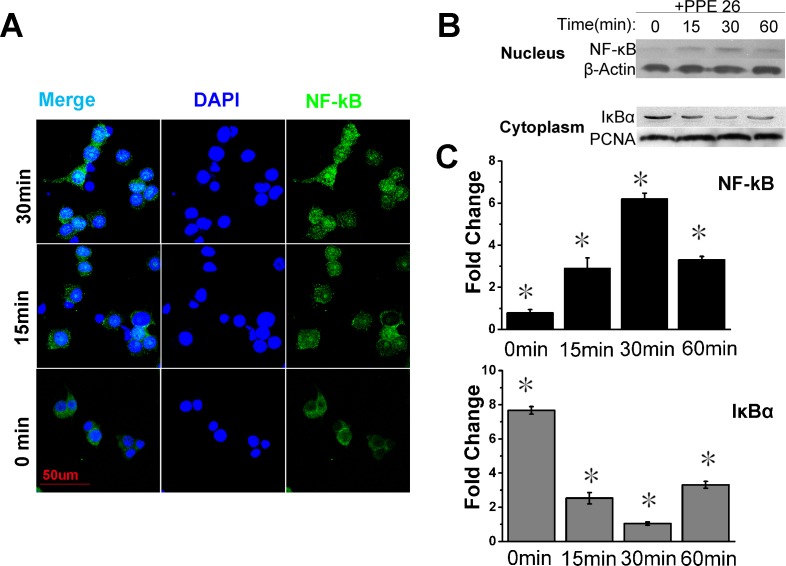
PPE26 affects NF-κB subunits subcellular location **A.**. Confocal microscopy examining the translocation of NF-κB subunits to the nucleus induced by PPE26. Scale bar, 50μm. **B.**. Western blot analysis showed that PPE26 translocates the NF-κB subunits from the cytoplasm to the nucleus and down-regulates IκBa expression in the nucleus. **C.**. Relative intensity of each band is expressed as the fold change compared to the value of the untreated controls. All data are expressed as the mean± SD (*n* = 3). *P < 0.05.

NF-κB is an important transcription factor that is involved in the induction of pro-inflammatory cytokines. Our MS analysis indicated that NF-κB was up-regulated 1.55-fold in the nucleus, which suggested the activation of the overall NF-κB-regulated signaling pathway. Thus, we examined the localization of NF-κB subunits in RAW264.7 cells treated with or without PPE26. Confocal microscopy demonstrated the translocation of NF-κB to the nuclei of PPE26-treated RAW264.7 cells. In unstimulated cells, NF-κB was present primarily in the cytoplasm (Figure 5A). Western blot analysis showed that stimulation of RAW264.7 cells with PPE26 induced the expression of nuclear NF-κB. The peak of nuclear NF-κB translocation occurred at 30 min and the nuclear expression of Iκ-Bα (the inhibitor of NF-κB) was also significantly down-regulated (Figure 5B and 5C).

### PPE26 increased the expression of co-stimulatory molecules and MHC molecules through TLR2

**Figure 6:**
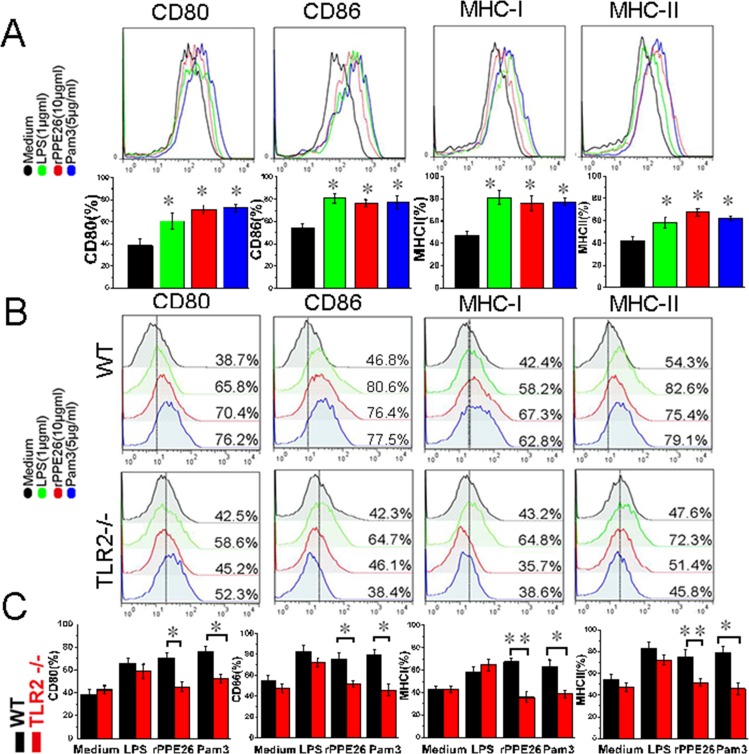
PPE26 enhances the expression of MHC molecules and co-stimulatory on macrophages via TLR2 **A.**. RAW264.7 cells were treated for 36 h with medium, PPE26 (10μg/ml), Pam_3_CSK4 (5 mg/ml) or LPS (1μg/ml). The expression of cell surface markers including CD80, CD86, MHC I and MHC II was examined by FACS analysis using the respective FITC or PE-linked mAbs. **B.**. Macrophages derived from WT and TLR2^−/−^ mice were treated for 36 h with medium, PPE26 (10μg/ml), Pam_3_CSK4 (5 mg/ml) or LPS (1μg/ml). The expression of cell surface markers including CD80, CD86, MHC I and MHC II was examined by FACS analysis using the respective FITC or PE-conjugated mAbs. **C.**. Bar graphs show the mean ± SD percentages of each surface molecule on macrophages in B, representing three independent experiments. *p < 0.05 and **p < 0.01.

Our MS data showed that H2-D1, H2-Aa, H2-L and H2-K1 were up-regulated 57%, 1.35-fold, 72% and 1.31-fold, respectively, and many proteins involved in antigen processing and presentation were also up-regulated (Table 2), suggesting that the PPE26 can fine-tune antigens presentation and processing (Supplementary Figure S9), and enhance the ability of macrophages to fully activate T cells. To clarify this finding, we examined the expression of co-stimulatory molecules and MHC molecules in PPE26-treated RAW264.7 cells by flow cytometry. The result demonstrated that 10μg/ml PPE26 significantly enhanced CD80, CD86, MHC I and II expression compared with control group (Figure 6A). Then, to investigate the role of TLR2 in mediating the up-regulation of PPE26-induced surface markers, we measured the expression of surface molecules in PPE26-treated macrophages from WT mice and TLR2^−/−^ mice using flow cytometry. As shown in Figure 6B and 6C, PPE26 remained capable of enhancing the expression of co-stimulatory and MHC molecules in the WT mice macrophages, although the expression of surface markers was strongly diminished in the TLR2^−/−^ mouse macrophages, which suggested the enhancement of the expression of cell surface markers in response to PPE26 stimulation is mediated by TLR2.

**Table 2:** Quantified Proteins Involved in MHC antigen processing and Presentation

Uniprot-ID	Location^a^	Gene Symbol	Protein Discription	H/L Ratio^b^	Coveragege (95%)	Unique Peptides	P value
P11499	cytosol	Hsp90b1	Heat shock protein HSP 90-beta	1.78	12.31	14	0.0025
Q3U2G2	cytosol	Hspa4	Heat shock 70 kDa protein 4	2.86	20.55	9	0.0221
Q3TBA3	cytosol	Tap1	Antigen peptide transporter 1	2.75	16.45	15	0.0378
Q3U9A3	cytosol	Tapbp	TAP binding protein	1.54	8.47	4	0.0157
P36371	cytosol	Tap2	Antigen peptide transporter 2	2.12	3.01	7	0.0247
P01897	cytosol	H2-L	H-2 class I histocompatibility antigen, L-D alpha chain	1.72	8.63	4	0.0108
Q31148	cytosol	H2-K1	H-2 class I histocompatibility antigen, K-B alpha chain	2.31	12.76	8	0.0381
G3UZP7	cytosol	H2-D1	H-2 class I histocompatibility antigen, D-P alpha chain	1.57	2.44	13	0.0256
Q860C1	cytosol	H2-Aa	H-2 class II histocompatibility antigen, A-S alpha chain	2.35	6.34	11	0.0318
P04441	cytosol	Cd74	H-2 class II histocompatibility antigen gamma chain	1.64	7.34	2	0.0161
Q5SUC3	cytosol	Canx	Calnexin	1.81	39.25	22	0.0201

a Proteins identified in cytosol or nuclear fraction.

b Proteins expression changes of PPE26-stimulated (H) vs CONTROL(L) macrophages.

### PPE26 drives a Th1-type immune response *via* TLR2-dependent macrophage activation

**Figure 7:**
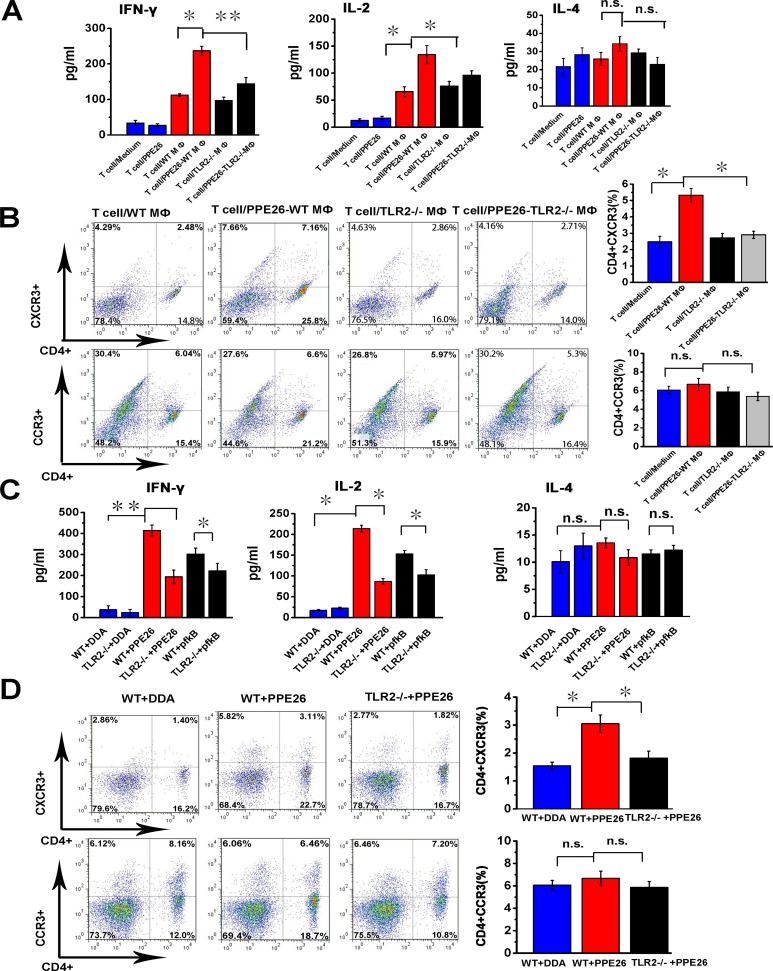
PPE26 induces a Th1-type immune response via TLR2-dependent macrophage activation **A.**. PPE26-activated T cells were obtained from splenocytes of C57BL/6 mice immunized with PPE26 (50 μg). Mouse peritoneal macrophages were isolated from TLR2^−/−^ or C57BL/6 mice. Macrophages were treated with 10μg/ml PPE26 protein for 24 h and then co-cultured with splenic T cells at a ratio of 1:10 for 3 days. The supernatant was used to measure the production of IFN-γ, IL-2 and IL-4 by ELISA. **B.**. T cells collected from A were stained with FITC-conjugated anti-CD4 mAbs, PE-conjugated anti-CXCR3 mAbs or PE-conjugated anti-CCR3 mAbs, and analyzed by flow cytometry. Histograms and bar graphs show the CXCR3^+^ or CCR3^+^ T cells in the PPE26-specific CD4^+^ T cells. **C.**. TLR2^−/−^ and C57BL/6 mice were immunized with 50 μg PPE26 mixed with DDA, 50 μg pfkB mixed with DDA or DDA alone. Splenocytes isolated from the immunized mice were stimulated with corresponding antigens (10μg/ml). The supernatants were used to measure the production of IL-2, IFN-γ and IL-4 by ELISA. **D.**. Lymphocytes collected from C were stained with FITC-conjugated anti-CD4 mAbs, PE-conjugated anti-CXCR3 mAbs or PE-conjugated anti-CCR3 mAbs, and analyzed by flow cytometry. Histograms and bar graphs show the CD4^+^/CXCR3^+^ or CD4^+^ / CCR3^+^ T cells. Values are means ± SD from three independent experiments. n.s. not significant *P < 0.05 and **P < 0.01.

To determine the effect of PPE26 stimulation on the interaction between macrophages and T cells, we performed a mixed lymphocyte reaction (MLR) assay using PPE26-specific T cells co-cultured with PPE26-pulsed macrophages or macrophages alone. ELISA analysis shown that T cells primed with PPE26-treated macrophages produced significantly higher levels of IFN-γ and IL-2 compared to T cells primed with untreated macrophages, whereas IL-4 secretion changed little (Figure 7A). Then, we investigated the expression of chemokine receptors CXCR3 and CCR3 using flow cytometry. As shown in Figure 7B, T cells co-cultured with PPE26-pulsed macrophages exhibited significantly increased CXCR3 expression compared to control group. In contrast, the expression of CCR3 in the presence of PPE26 treatment remained unaffected. Importantly, T cells co-cultured with PPE26-pulsed C57BL/6 macrophages showed robust IFN-γ and IL-2 immune responses and higher expression of CXCR3 when compared to those co-cultured with PPE26-pulsed TLR2^−/−^ macrophages (Figure 7A and 7B). These findings demonstrated that PPE26-stimulated macrophages induce the proliferation of naïve T cells towards a Th1 phenotype in vitro.

Next, to investigate whether PPE57 induced a Th1-type immune response via TLR2-mediated macrophage activation, we injected PPE26 into wild-type or TLR2^−/−^ mice and measured IFN-γ, IL-2 and IL-4 secretion from T cells as well as the expression of CXCR3 and CCR3. As demonstrated in Figure 7C and 7D, PPE26 increased IFN-γ and IL-2 production, and enhanced CXCR3 expression in T cells in WT mice; in contrast, IL-4 secretion and CCR3 expression remained unchanged. In TLR2-deficient mice, no alteration in the IFN-γ, IL-2 and IL-4 secretion, or CXCR3 and CCR3 expression induced by PPE26 treatment was detected. The results suggested that PPE26 appears to activate macrophages and induce Th1-type immune response.

### Recombinant mycobacterium bovis BCG expressing PPE26 enhances the Th1 cell-mediated response and promotes the development and maintenance of effector/memory T cells

**Figure 8:**
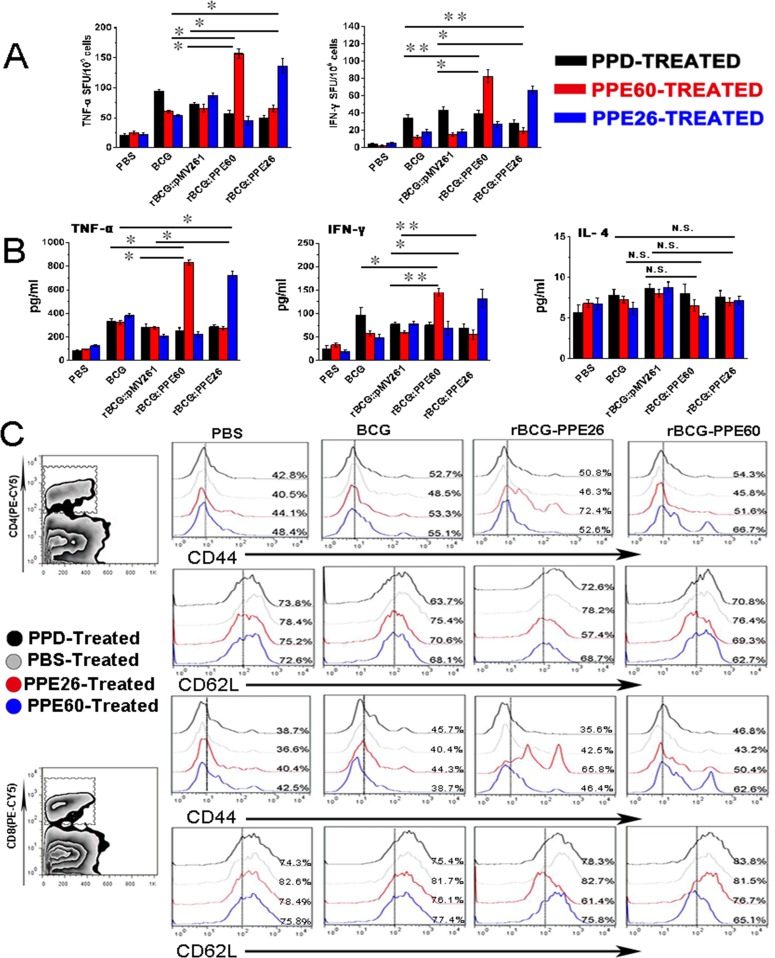
Recombinant BCG expressing PPE26 enhances the Th1-type immune response and induces effector/memory T cell proliferation **A.**. Splenocytes were isolated from C57BL/6 mice immunized with PBS, BCG, rBCG::pMV261, rBCG::PPE60, or rBCG::PPE26. Then, splenocytes were stimulated with PPD (10μg/ml), PPE60 (10μg/ml) or PPE26 (10μg/ml) for 36 h, and the cellular immune response was measured by ELISPOT assay. **B.**. Cytokine production by mouse spleen cells was assayed by a sandwich ELISA after stimulation of the cells with PPD (10μg/ml), PPE60 (10μg/ml), or PPE26 (10μg/ml). **C.**. Splenocytes were isolated from C57BL/6 mice immunized with PBS, BCG, rBCG::pMV261, rBCG::PPE60, or rBCG::PPE26. Splenocytes were stimulated with PPD (10μg/ml), PPE60 (10μg/ml), or PPE26 (10μg/ml) or PBS for 36 h. The cells were stained with FITC-conjugated anti-CD4 mAbs or FITC-conjugated anti-CD8 mAbs, PE-conjugated anti-CD62L mAbs and PE-Cy5-conjugated anti-CD44 mAbs, and then analyzed by flow cytometry. Values are means ± SD from three independent experiments. n.s. not significant *P < 0.05 and **P < 0.01.

Our data indicated that PPE26 had good potential as a vaccine for its effective induction of both cell-mediated and humoral immune responses. Thus, we constructed recombinant BCG expressing PPE26 (rBCG::PPE26) and compared the immunogenicities between rBCG::PPE26 and BCG. Figure 8A illustrated that immunization with this strain induced stronger PPE26-specific IFN-γ and TNF-α activity than those in the control group immunized with BCG. Moreover, rBCG::PPE26 significantly increased the Th1 cytokines IFN-γ and TNF-α production in splenocyte cultures comparable to those elicited by control group, wheras no alteration in IL-4 secretion was observed (Figure 8B).

To assess whether the PPE26-induced macrophages activation is reflected in the ability to specifically stimulate CD4^+^ and CD8^+^ splenic T cells isolated from rBCG::PPE26 immunized mice, we analyzed the expression of CD62L and CD44 on CD4^+^ and CD8^+^ splenic T cells using flow cytometry. As shown in Figure 8C, PPE26 was found to induce the formation of effector/memory T cells by displaying significantly down-regulated CD62L and up-regulated CD44 expression on both CD4^+^ and CD8^+^ T spleen cells, demonstrating that rBCG::PPE26 could effectively promote the development of effector/memory CD4^+^/CD8^+^CD44^high^CD62L^low^ T cells. Taken together, our findings indicated that rBCG::PPE26 enhances the Th1 cell-mediated response, induces the development of effector/memory T cells, and may serve as a potential vaccine against *M. tuberculosis*.

## DISCUSSION

PE/PPE family proteins of *M. tuberculosis* play a critical role in generating antigenic variation and evasion of host immune response. Understanding the functional characterization of mycobacterial PE/PPE proteins is essential for the comprehension of the host-pathogen interaction and the design of prospective vaccine candidates. However, little is known about the functional roles and the underlying mechanisms of PE/PPE proteins of *M. tuberculosis*.

In this study, to clarify the link between PPE26 and the host response, we analyzed the proteome changes during host response to PPE26 stimulation by iTRAQ subcellular quantitative proteomics. The results demonstrated that TLR2 was increased 54% in rBCG::PPE26 infected macrophages compared to BCG infected macrophages, while other TLR family members were not detected. As the key adapter protein essential for the signaling of antigens via the TLR family (except TLR3) [33, 45], Myd88 was shown to be increased by 1.88-fold in the cytosol. Moreover, MAPKs-associated kinases and NF-κB were also significantly up-regulated. MS data suggested that PPE26 may activate the TLR2-MyD88-mediated signaling necessary for macrophages activation. The concurrent biological validations also revealed that PPE26 could directly bind to TLR2 and activate MAPKs and NF-κB pathway. Therefore, the results indicate that PPE26 is a novel macrophage activation-inducting antigen through triggering a cross-talk connection between multiple pathways downstream of TLR2.

Different PE/PPE proteins appear to trigger different downstream TLR2 signaling cascades and induce the production of either pro or anti-inflammatory cytokines [46, 47]. PPE18 and PPE34 could activate the TLR2-MAPK pathway and induce the production of IL-10, which is an anti-inflammatory cytokines beneficial for the survival of *M. tuberculosis* [26, 27]. However, a few PE/PPE proteins are capable of triggering APC to secret pro-inflammatory cytokines. In this study, we observed that PPE26 could activate macrophages by binding to TLR2 and induce higher levels of TNF-α, IL-6, and IL-12 p40. TNF-α is important for granuloma formation and the clearance of mycobacterium [48]. IL-12 p40 was previously reported to promote the production of IFN-γ and drive the protective Th1 immune response against *M. tuberculosis* bacilli [49]. Furthermore, the induction of these cytokines was higher in macrophages from wild-type than those from TLR2^−/−^ mice. These findings demonstrate that PPE26 may be a potent TLR2 agonist and can induce the secretion of pro-inflammatory cytokines.

T cells require two signals to become fully activated. Antigen-specific signal is provided through the T cell receptor which interacts with peptide-MHC molecules on the membrane of antigen presenting cells (APC) [50]. Our MS data showed that H2-D1, H2-Aa, H2-L and H2-K1 were up-regulated 57%, 1.35-fold, 72% and 1.31-fold, respectively. As the key components of the multi-protein peptide loading complex, TAP and TAPBP were also significantly up-regulated. Moreover, PPE26 significantly increased the expression of MHC I and II in RAW264.7, suggesting that PPE26 promotes the antigen processing and presentation, and enhances the antigen-response signal. Co-stimulatory signal is necessary for T cell activation and survival. CD80 and CD86 are the mainly co-stimulatory molecules expressed on APC, and can bind to TCR CD28 to provide co-stimulatory signaling [51, 52]. In our study, we found that PPE26 could dramatically increase the expression of CD80 and CD86 on macrophages. Taken together, our results indicate that PPE26 is capable of promoting the interaction between macrophages and T cells, and sustaining the two signals required for T cell activation.

Protection against *M. tuberculosis* infection depends on the rapid and continued generation of Type 1 cytokines (especially IFN-γ and IL-2), which activate phagocytes to constrain the intracellular mycobacterial pathogen [53]. IFN-γ has a strong effect on the controlling/killing intracellular bacterium, the IFN-γ receptor deficiency increased the susceptibility of patients to mycobacterial infections [54–56]. IL-2 amplifies memory/effector T cells functions by increasing antigenic sensitivity and improving memory capacity [57]. However, Th2 cells generate Type 2 cytokines (IL-4, IL-10) and suppress Th1-cell mediated immune response. Our results demonstrated that PPE26 significantly increased the production of IFN-γ and IL-2 in CD4^+^ T cells through the MLR analysis in vitro, suggesting that PPE26 can enhance the Th1 immune response and promote effector T cell functions. Chemokine receptors expression during T cell activation shapes the immune response by preferential homing of distinct T cells subsets. CCR5 and CXCR3 are preferentially expressed on Th1 cells, whereas Th2 cells mainly express CCR3 and CCR4 [58, 59]. In this context, we observed that PPE26-induced macrophages increased the expression of CXCR3 in CD4^+^ T cells, while the CCR3 level remained at the baseline. Together, our data indicate that PPE26 may regulate adaptive immunity by directing T cell immune responses towards Thl polarization.

Attempts to replace conventional BCG with recombinant BCG to achieve stronger protective efficacy and/or increased safety have been the focus of genetic engineering experiments [2]. In this study, we constructed the rBCG::PPE26 strain and evaluated its immunogenicity. Our results showed that immunization with this strain induced stronger antigen-specific IFN-γ and TNF-α activities as determined by ELISPOT assay, and higher levels of antigen-specific CD4^+^ and CD8^+^ T-cell responses compared to the group immunized with BCG. Likewise, rBCG::PPE26 significantly increased the production of IFN-γ, TNF-α, and IL-2 in splenocyte cultures compared to those elicited by BCG. Our findings demonstrate that rBCG::PPE26 enhances the Th1 cell-mediated response, and may serve as a potential vaccine against *M. tuberculosis*.

Immune control of *M. tuberculosis* depends on the rapid proliferation of effector memory T cells. These cell subpopulations capable of producing IFN-γ are considered to be the key components of acquired immunity and the basis for successful vaccination against TB [60]. Chief cell surface molecules include the lymph node-homing molecule CD62L and memory T cell proliferation marker molecule CD44. Effector/memory T cells express the CD44^high^CD62L^low^ surface phenotype [61, 62]. Previously, CD4^+^ or CD8^+^ T cells with down-regulated CD62L and up-regulated CD44 expression were reported to accumulate at the site of infection [63]. Moreover, IFN-γ producing cells could be described as cells expressing the CD44^high^ and CD62L^low^ phenotype. These cells enable the host to react quickly and control a recognized pathogen if encountered again [64]. Therefore, to augment the proportion of specific T cells with the CD44^high^CD62L^low^ phenotype is of great importance in the context of vaccine designs and immunization strategies against TB. Our data showed that a population of CD4^+^ or CD8^+^/CD44^high^CD62L^low^ T cells was specifically generated from splenic T cells in rBCG::PPE26 immunized mice, suggesting that PPE26 is a specific recall antigen to trigger Th1-mediated immune responses. Taken together, PPE26 may link adaptive immunity and promote the formation and proliferation of effector memory T cells.

Collectively, our work indicates that PPE26 can directly bind to TLR2 and induce pro-inflammatory response by initiating a cross-talk of multiple pathways. PPE26 effectively modulates innate and adaptive immune responses through polarizing the development of T cells towards a Th1 phenotype. Moreover, PPE26 can augment the proportion of effector/memory T cells with a CD4^+^ or CD8^+^/CD44^high^CD62L^low^ phenotype in rBCG::PPE26 immunized mice. These novel findings demonstrate that PPE26 is a good antigen for the rational design of new strategies to prevent many chronic disease caused by *M. tuberculosis*.

### Key Messages

PPE26 triggers the cross-talk of multiple pathways involved in the host response, as revealed by an iTRAQ-based subcellular quantitative proteomics approach.

PPE26 induces cytokine production and up-regulates the function of mouse macrophages through TLR2.

PPE26 drives a Th1 immune response via TLR2-mediated macrophage functions.

Recombinant BCG over-expressing PPE26 induces stronger antigen-specific IFN-γ activity and higher levels of Th1 cytokines, causing Th1-polarized T-cell expansion.

## MATERIALS AND METHODS

### Mice and cell lines

C57BL/6 mice were purchased from the Animal Center of Slaccas (Shanghai, China). TLR2^−/−^ and TLR4^−/−^ mice 6-8 weeks of age were obtained from Model Animal Research of Nanjing University (Nanjing, China). All mice were housed under specific pathogen-free conditions in the Animal Center of the School of Life Science of Fudan University. The experimental procedures followed the Guidelines for the Care and Use of Laboratory Animals from the National Institutes of Health and were approved by the Animal Care and Use Ethical Committee of Fudan University. The RAW264.7 cell line was purchased from the Cell Bank of the Chinese Academy of Sciences (Shanghai, China). Cells were cultured in Dulbecco's modified Eagle's medium (DMEM) (Gibco, Grand Island, NY, USA) supplemented with 10% fetal bovine serum (FBS), penicillin (100U/ml) and streptomycin (100 mg/ml) and maintained at 37°C in a humidified incubator (5% CO_2_).

### Cloning and expression of recombinant PPE26

The PPE26 gene was amplified using PCR based on the genomic NA sequence of M. tuberculosis H37Rv with specific forward and reverse primers (Supplementary Table 1). After treatment with the BamH1 and EcoR1 restriction enzymes, the PCR product was sub-cloned into the expression vector Pet28a and transformed into competent *Escherichia coli* BL21. Recombinant PPE26 was prepared by the induction of bacterial cells by 0.5 mM IPTG at 37°C for 4 h. The harvested bacteria were suspended in 20 mM Tris-HCl (pH 8.0), 0.5 M NaCl, and 20 mM imidazole and lysed by sonication. Recombinant PPE26 was purified using a HIS-Select^®^ Nickel Affinity Gel (Sigma-Aldrich, St. Louis, MO, USA) following the manufacturer's instructions and identified by immunoblot using anti-His antibodies. Recombinant protein was treated with Pierce High Capacity Endotoxin Removal Resin (Pierce, USA) in accordance with the user instructions to eliminate endotoxins. The recombinant protein was quantified with a bicinchoninic acid (BCA) protein assay kit (Pierce, Rockford, IL, USA) and frozen at −80°C.

### Construction of rBCG::PPE26

The PPE26 gene was amplified using PCR based on the genomic NA sequence of *M. tuberculosis* H37Rv with specific forward and reverse primers (Supplementary Table 1). After treatment with the BamH1 and EcoR1 restriction enzymes, the PCR product was sub-cloned into the expression vector pMV261, generating pMV261::PPE26. The constructs were electroporated into *Mycobacterium bovis Bacillus Calmette-Guérin* (BCG). The selected rBCG::PPE26 transformants were cultured in Middlebrook 7H9 with 10% oleic albumin dextrose catalase (OADC) containing 50μg/ml kanamycin. The rBCG::PPE26 was identified by immunoblotting using anti-PPE26 mouse polyclonal (Supplementary Figure S1A).

### Infection of RAW264.7 macrophages

RAW264.7 cells were maintained in DMEM supplemented with 10% FBS at 37°C in 5% CO2. BCG or rBCG::PPE26 was pelleted during the exponential growth phase, washed twice with PBS and resuspended. Prior to infection, a bacterial single-cell suspension was prepared by vortexing the cells with glass beads, followed by centrifugation at low speed and passage through a 5μm syringe filter to remove bacterial aggregates[29]. The RAW264.7 cells were infected with bacteria at a multiplicity of infection (MOI) of 10 for 4 h at 37°C in a 5% CO_2_ environment, after which time the cells were washed three times with PBS. A concentration of 200 μg/ml gentamicin (Sigma, St. Louis, MO, USA) was added to the cells for 2h to remove the extracellular bacteria. The number of colony-forming units (CFUs) recovered from the macrophages was determined by plating the bacteria onto 7H10 agar.

### Subcellular fractionation

Cytosolic and nuclear fractions were prepared using the NE-PER Nuclear and Cytoplasmic Extraction kit (Pierce, Rockford, IL, USA) following the manufacturer's instructions. Briefly, cells were harvested and washed twice with PBS. The cells were suspended in buffer A with a protease inhibitor, and incubated on ice for 10 min. The supernatant was isolated by centrifugation (10min, 6000rpm). The remaining was suspended in buffer B on ice for 30min. The soluble fractions were separated by centrifugation (15 min, 14000 rpm). The procedure is shown in Supplementary Figure 2. The protein concentrations were determined using the bicinchoninic acid assay (BCA) protein assay kit (Pierce, Rockford, IL, USA).

### iTRAQ labeling

Protein labeling was performed as described previously [65]. Briefly, the desalted samples were firstly mixed with 30μl of SDT buffer [4% SDS, 100 mM DTT, and 150 mM Tris-HCl (pH 8.0)]. UA buffer [8 M urea and 150 mM Tris-HCl (pH 8.0)) was used to remove the detergent and DTT by repeated ultrafiltration (Microcon units, 30 kDa). After the samples were incubated with 100μl of 0.05 M iodoacetamide in UA buffer for 20 min in the dark, the protein suspensions were digested with 2μg of trypsin in 40μl of DS buffer overnight at 37°C. Finally, the peptides were labeled using the 4-plex iTRAQ reagent according to the manufacturer's instructions (Applied 245 Biosystems). The cytoplasmic samples were labeled as 113(control) and 115 (infection), and the nuclear samples were labeled as 116 (control) and 117 (infection).

### SCX-based fractionation and LC-MS/MS analysis

SCX chromatography was performed as described previously [66]. The peptides were fractionated on a PolySULFOETHYL A column (200 Å, 5μm, 200 × 2.1 mm) (PolyLC, Columbia, MD, USA) using an Agilent 1200 LC system (Agilent Technologies). Peptide fractions were collected using a linear gradient of solvent B (350 mM KCl in solvent A, pH 2.8) over 70 min at a flow rate of 300 μl/min. Subsequently, the desalted peptide samples were analyzed using a Q Exactive mass spectrometer coupled to an Easy nLC (Proxeon Biosystems, now Thermo Fisher Scientific). MS data were dynamically acquired by choosing the most abundant precursor ions from the survey scan (300-1800 m/z) for HCD fragmentation. The dynamic exclusion duration was 60 s. Survey scans were acquired at a resolution of 70,000 at 200 m/z. The resolution for HCD spectra was set to 17,500 at 200 m/z. The normalized collision energy was 30eV.

### Proteomics data analysis

Protein identification and quantification were performed with MaxQuant version 1.2.0.18. The data were searched using the Andromeda search engine against the IPI mouse database. Parameters for the searches were as follows: trypsin = enzyme; missed cleavage = 1; variable modification: oxidation (M); peptide mass tolerance = 20 ppm; MS/MS tolerance = 0.1 Da; FDR≤1%; iTRAQ modification at the N-terminus of the peptide and lysine. Relative expression pattern of proteins was determined based on the relative intensities of reporter ions of the peptides. Criteria to select the confident list of differential proteins were set to ≥2 peptides, single peptide with multiple PSM values and p-value ≤ 0.05.

### Functional clustering and network analysis

The quantified proteins were submitted to DAVID (http://david.abcc.ncifcrf.gov/) to obtain their known biological processes and molecular functions. Proteins involved in signaling pathways were categorized by PANTHER (http://www.pantherdb.org/). A network was constructed by STRING (http://string-db.org/) according to their categorized functions. The links in the network were edited by Cytoscape (http://www.cytoscape.org/).

### Measurement of cytokines

Sandwich ELISA kits were used to detect TNF-α, IL-6, and IL-12p40 levels in culture supernatants. Briefly, RAW264.7, macrophages from WT, TLR2^−/−^, or TLR4^−/−^ mouse were cultured in 24-well plates and then were treated with medium, PPE26(0.1-10μg/ml), Pam_3_CSK4 (5 mg/ml), isotype IgG (50μg/ml), proteinase K (50μg/ml) or proteinase K (50μg/ml) + PPE26 (10μg/ml) or LPS (1μg/ml) for 24 h. Cytokine levels of TNF-α, IL-6, and IL-12p40 in the culture media were measured as recommended by the manufacturer (BioLegend, San Diego, CA, USA). The cytokine assays were performed by measuring the absorbance at 450 nm with a microplate reader. The modified pharmacological inhibitor experiment was designed as previously described [20]. Briefly, block of the MAPK signaling pathway experiments involved the pretreatment of RAW264.7 cells with inhibitors to p38 (SB203580, 10μM), ERK (U0126, 10μM) or JNK (SP600125, 10μM) for 1 h at 37°C, followed by incubation with PPE26 for 36 h at 37°C. The IgG isotype control A and proteinase K were purchased from Sigma-Aldrich (St. Louis, MO, USA).

### RT-PCR and quantitative RT-PCR analysis

RAW246.7 cells were seeded in 24-well plates for 12 h and then treated with medium, protease K (50μg/ml), PPE26 (10μg/ml) or Pam_3_CSK4 (5mg/ml) for 24 h. The cells were harvested and rinsed twice with PBS. 1 ml/well of TRIzol (Invitrogen, Carlsbad, CA, USA) was added to each tube and cultured at room temperature for 5 min. The cells were mixed thoroughly two times with chloroform:isoamyl alcohol (1:1), and centrifuged at 10000rpm for 15min, then rinsed with 75% ethanol (DEPC-treated water) and dissolved in DEPC-treated water. First-strand cDNA was synthesized by reverse transcription using the PrimeScript RT reagent Kit with gDNA Eraser (TaKaRa Biotechnology, Japan). The target genes were amplified by conventional methods and appropriate cDNA templates. RNA levels of the analyzed genes were normalized to the amount of β-actin present in each sample. All primers (Supplementary Table 1) were synthesized by Sangon (Shanghai, China).

### Immunoprecipitation

C57BL/6 mouse macrophages were treated with PPE26 (10μg/ml) for 6 h and lysed with RIPA lysis buffer (Sangon, China). After pre-clearing with protein A/G sepharose beads (Santa Cruz, CA, USA) for 2 h, the mixture of cell lysates and bead was centrifuged at 10,000 x g for 5 min at 4°C. The supernatant was incubated with anti-TLR2, anti-TLR4 or anti-PPE26 overnight at 4°C, then the Ab-bound proteins were pull down using protein A /G beads for 6 h at 4°C. The beads were harvested, washed, and boiled in 5x sample buffer for 5 min. The proteins were separated on 10% SDS-PAGE and probed with anti-TLR2, anti-TLR4 (BioLegend, CA, USA), and anti-His Abs (Santa Cruz, CA, USA) as indicated, followed by incubation with HRP-conjugated mouse anti-rat or rabbit anti-mouse secondary IgG secondary Abs. Target bands were visualized using the ECL reagent (Thermo Fisher Scientific, MA, USA).

### Toll-like receptor binding assays

WT, TLR2^−/−^ and TLR4^−/−^ mouse macrophages were incubated with PPE26-His (10μg/ml) for 1 h at 37°C. The cells were fixed in 4% PFA for 15 min, and then permeabilized in PBST (0.1% Triton X-100) for 15 min. After blocked with 5% BSA in PBST for 2 h, the cells were incubated with anti-TLR2 (1:200), anti-TLR4 (1:200) and anti-His Abs (1:500) overnight at 4°C. The cells were incubated with the Alexa Fluor^®^568 donkey anti-mouse IgG (Santa Cruz, CA, USA) or Alexa Fluor^®^488 donkey anti-rabbit IgG (Santa Cruz, CA, USA) secondary antibodies for 2 h in the dark room and then stained with 0.5 g/ml DAPI (Santa Cruz, CA, USA) for 5 min at room temperature. Between each staining step, the cells were washed three times with PBS for 5min. Finally, the cells were mounted onto slides using ProLong^®^ Gold Antifade Mountant (Thermo Fisher Scientific, MA, USA) and observed using a 63X oil objective on a Zeiss LSM 710 microscope (Carl Zeiss, Germany). Images were acquired by the LSM710 Meta software and processed using image J (1.4.4).

### Western blot analysis

RAW264.7 cells or macrophages from C57BL/6, TLR2^−/−^ or TLR4^−/−^ mice were stimulated with PPE26 (10μg/ml) for indicated time, and lysed with cell lysis buffer supplemented with a proteinase inhibitor mixture (Roche Molecular Biochemicals, Indianapolis, IN, USA). Cell pellets were processed using the NE-PER Nuclear and Cytoplasmic Extraction kit (Pierce, Rockford, IL, USA) following the manufacturer's instructions. Equal amounts of proteins were separated on 10% SDS-PAGE and then transferred electrophoretically to PVDF membranes from Millipore (MA, USA). After treated with blocking buffer, the membranes were incubated with primary Abs overnight at 4°C, including rabbit anti-ERK2, rabbit anti-p38, rabbit anti-JNK, rabbit anti-phospho-ERK1/2, rabbit anti-phospho-p38, rabbit anti-phospho-JNK, rabbit anti-phospho-IκB-α, rabbit anti-NF-κB p65, rabbit anti-PCNA or rabbit anti-β-actin (Santa Cruz, CA, USA). After washed with TBST buffer, the membranes were incubated with the HRP-conjugated secondary Abs for 2 h at room temperature. Target proteins were visualized using the Pierce ECL Western Blotting Substrate (Pierce, Rockford, IL, USA).

### Flow cytometric analysis

RAW264.7 or macrophages from WT or TLR2^−/−^ mouse were incubated with PPE26 (10μg/ml), Pam_3_CSK4 (5 mg/ml), or LPS (1μg/ml) for 36 h. Then, the cells were harvested and washed with prechilled PBS, followed by centrifugation at 1000 x g for 10 min at 4°C. The cells were treated with Fc Block (1:100) (BD Pharmingen, CA, USA) in PBS supplemented with 1% BSA and incubated with PE-conjugated anti-mouse CD86, FITC-conjugated anti-mouse CD80, PE-conjugated anti-mouse H-2κB for mouse macrophages, or FITC-conjugated anti-mouse I-A/I-E (BD Pharmingen, CA, USA) on ice for 1 h in the dark room. The cells were resuspended in 500μl PBS and analyzed using a flow cytometer (Becton Dickinson, USA). The data were analyzed using the Cell-Quest data analysis software (10000 events per sample) and Flow4J.

### Mixed lymphocyte reaction assay and Analysis of the Th1 response *in vivo*

TLR2^−/−^ or C57BL/6 mice were immunised subcutaneously three times over a 2-week period with 50 μg of PPE26 formulated with dimethyldioctadecylammonium (DDA) adjuvants (Sigma, Louis, MI, USA). PPE26-activated T cells were isolated from total mononuclear cells prepared from C57BL/6 mice using a MACS column. Mouse peritoneal macrophages were isolated from TLR2^−/−^ or C57BL/6 mice as previously described [29]. Briefly, the mice were euthanized, and the peritoneal cavities were flushed with 5 ml of ice-cold RPMI 1640 medium without FBS. Peritoneal cells were enriched by centrifugation, seeded into six-well plates in RPMI 1640 containing 10% FBS and incubated overnight at 37°C. Non-adherent cells were removed, and the adherent cells were washed twice with PBS and then treated with 10μg/ml of PPE26 for 24 h. T cells and macrophages were co-cultured at a 1:10 ratio for 72 h at 37°C. Cytokines in the culture media (IFN-γ, IL-2 and IL-4) were measured by ELISA. Harvested T cells were stained with FITC-conjugated anti-CD4 mAbs, PE-conjugated anti-CCR3 mAbs or PE-conjugated anti-CXCR3 mAbs (BD Pharmingen, San Diego, CA, USA) and analyzed by flow cytometry.

TLR2^−/−^ or C57BL/6 mice were injected subcutaneously with equal amounts (50 μg) of DDA, DDA+PPE26 or DDA+ pfkB (Rv2029c) (an irrelevant mycobacterium antigen) to immunize the mice. After three administrations, the mice were euthanized and lymphocytes were isolated from spleen cells using Lymphocyte-M density-gradient centrifugation (Cedar Lane Lab, Burlington, NC, USA) following the manufacturer's instructions. The cells were treated with equal amounts (10 μg) of DDA, PPE26 or pfkB for 36 h. Lymphocytes were stained with FITC-conjugated anti-CD4 mAbs, PE-conjugated anti-CCR3 mAbs or PE-conjugated anti-CXCR3 mAbs, and analyzed by flow cytometry. The concentrations of IL-2, IFN-γ and IL-4 in each supernatant sample were measured by ELISA.

### Immunization of experimental animals

Groups of 12 C57BL/6 mice were immunized subcutaneously with 5×10^6^ CFUs of BCG, rBCG::PMV, rBCG::PPE26 or rBCG::PPE60 (positive control) in 100μl of PBS. Vaccine control mice received the Pasteur strain BCG. Lymphocytes were isolated from spleen cells 12 weeks post-vaccination using Lymphocyte-M density-gradient centrifugation. IFN-γ and TNF-α ELISPOT kits (U-CyTech Bio-sciences, Netherlands) were used to determine the relative number of IFN-γ- or TNF-α-positive cells in the single cell suspensions following the manufacturer's instructions. The spot-forming units (SFU) were counted using a dissecting microscope. The levels of IFN-γ, IL-4 and TNF-α were measured by ELISA. Responder T cells were isolated from total mononuclear cells prepared from the immunized mice using a MACS column. Cells were treated with corresponding antigens for 36 h, stained with FITC-conjugated anti-CD4 mAbs, or FITC-conjugated anti-CD8 mAbs, PE-conjugated anti-CD62L mAbs and PE-cy5-conjugated anti-CD44 mAbs and analyzed by flow cytometry. All mAbs come from BD Pharmingen (San Diego, CA, USA).

### Statistical analysis

Results were calculated as the mean ± SD of triplicate experiments. Statistical analysis was conducted using a one-way ANOVA followed by Tukey's test using the origin8.0 software (Origin Lab, USA). For all tests, *p* ≦ 0.05 was considered statistically significant.

## SUPPLEMENTARY MATERIAL FIGURES AND TABLES















## Abbreviations

−/−: knockout
BCG: Bacille Calmette Guerin
TLR: Toll-like receptors
CB: coomassie blue
CD62L: CD62 ligand
TB: tuberculosis
M. tuberculosis: Mycobacterium tuberculosis
PBST: PBS containing 0.1% Tween-20
TRIF: Toll/IL-1R homology domain-containing adapter-inducing IFN-γ
PAMP: Pathogen-associated molecular patterns
LPS: Lipopolysaccharides
CFU: colony-forming unit
IRFs: interferon regulatory factors
SDS-PAGE: sulphate-polyacrylamide gel electrophoresis
ELISPOT: enzyme-linked immune spot assay
ELISA: enzyme-linked immune sorbent assay
MHC: histocompatibility complex
IFN-γ: Interferon gamma
CXCR3: Chemokine receptor
CCR3: C-C chemokine receptor type 3
CXCR5: Chemokine receptor CXCR5
CCR4: C-C chemokine receptor type 4
Pam3: Pam3CSK4.
