# Supplementary material for: PPE26 induces TLR2-dependent activation of macrophages and drives Th1-type T-cell immunity by triggering the cross-talk of multiple pathways involved in the host response
Source: Oncotarget. 2015 Oct 2;6(36):38517–37. doi: 10.18632/oncotarget.5956 (PMC4770718; doi:10.18632/oncotarget.5956)
Supplement: Supplementary file 1 [file oncotarget-06-38517-s001.pdf]

PPE26 induces TLR2-dependent activation of macrophages and drives Th1-type T-cell immunity by triggering the cross-talk of multiple pathways involved in the host response

Supplementary Material

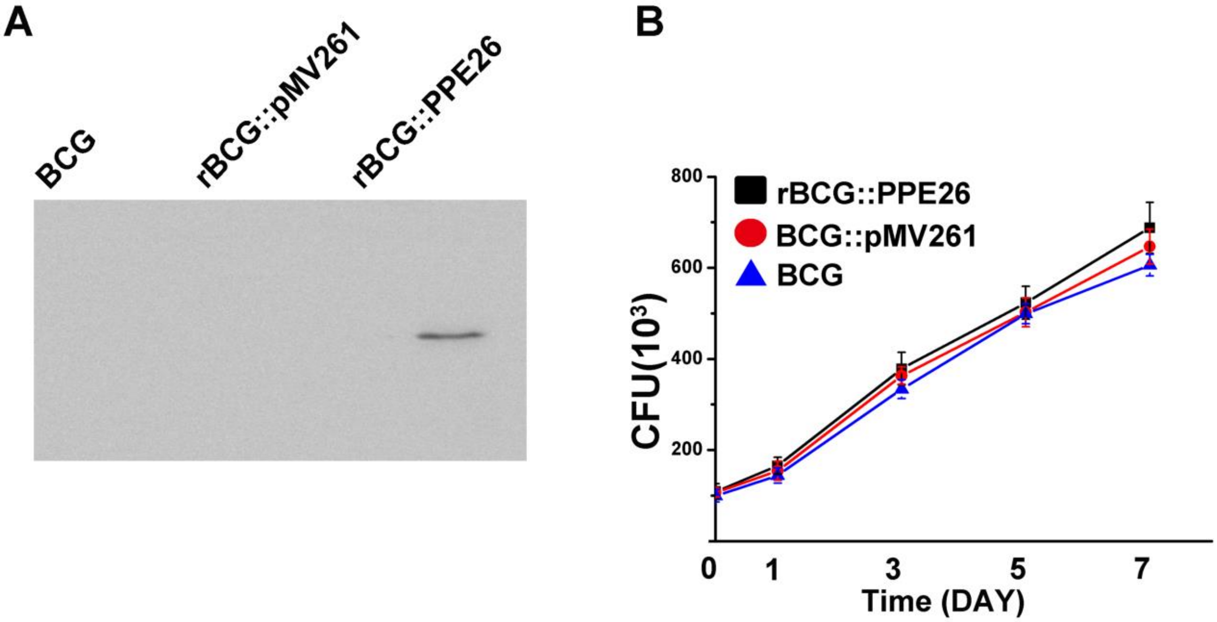

Supplementary Figure S1: The effect of PPE26 on the growth of mycobacteria.

(A). rBCG::PPE26 lysates were analyzed for expression by immunoblotting with antibodiesrecognizing PPE26. Lane 1. BCG， Lane 2. rBCG::pMV261， Lane3. rBCG::PPE26. **(B).** Intracellular replication of the BCG, rBCG-PMV and rBCG-PPE57 strains. The infection experiment was performed at an MOI of 0.5, and the data were generated from three independent experiments.

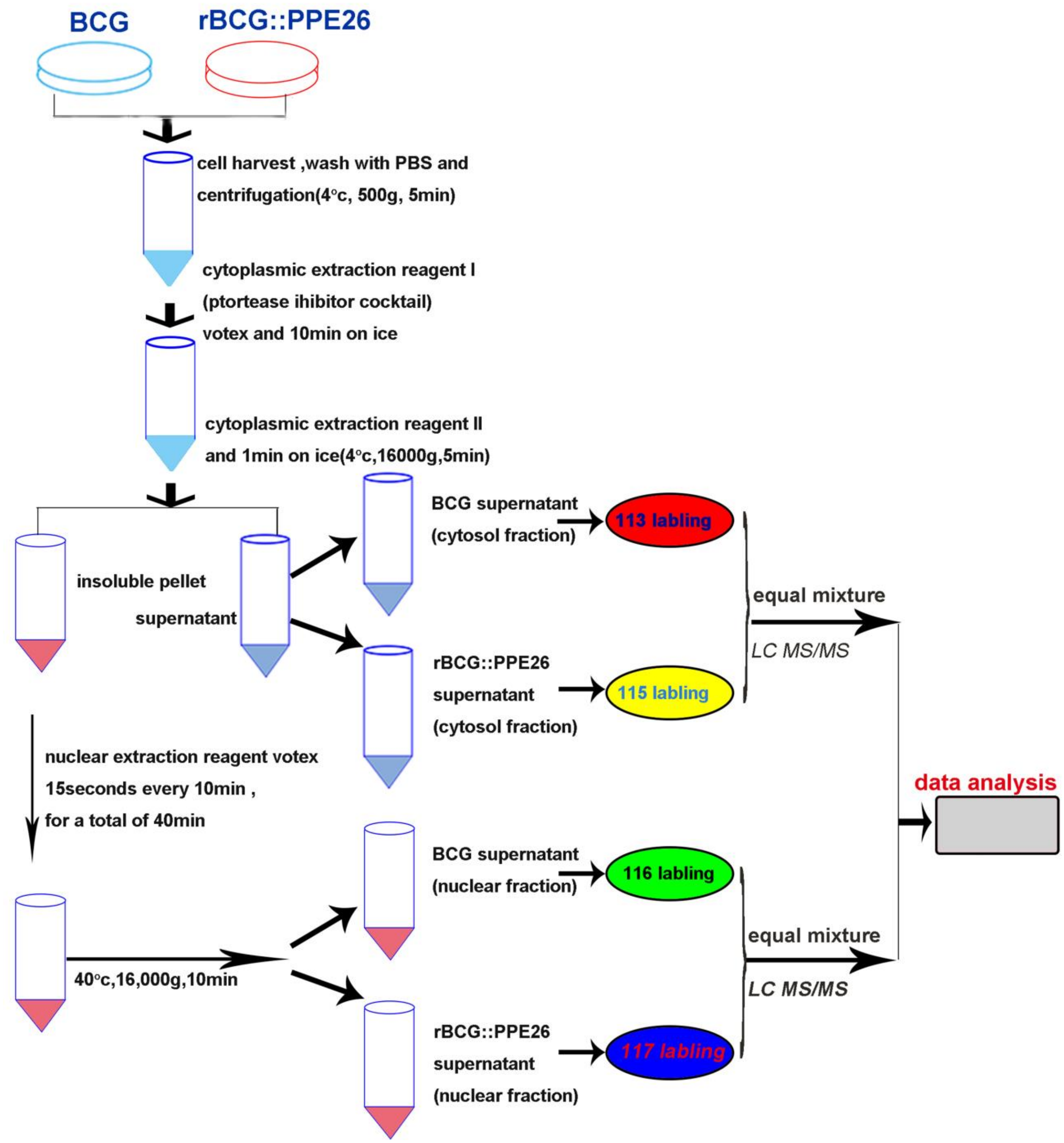

Supplementary Figure S2: Schematic describing the iTRAQ quantitative proteomic approaches for protein extraction.

Supplementary Figure S3: the MS/MS spectrum and quantification information for typical proteins. Insets shows relative intensities of reporter ions of typical proteins, the cytoplasmic samples were labeled as 113(control) and 115 (infection), and the nuclear samples were labeled as 116 (control) and 117 (infection).

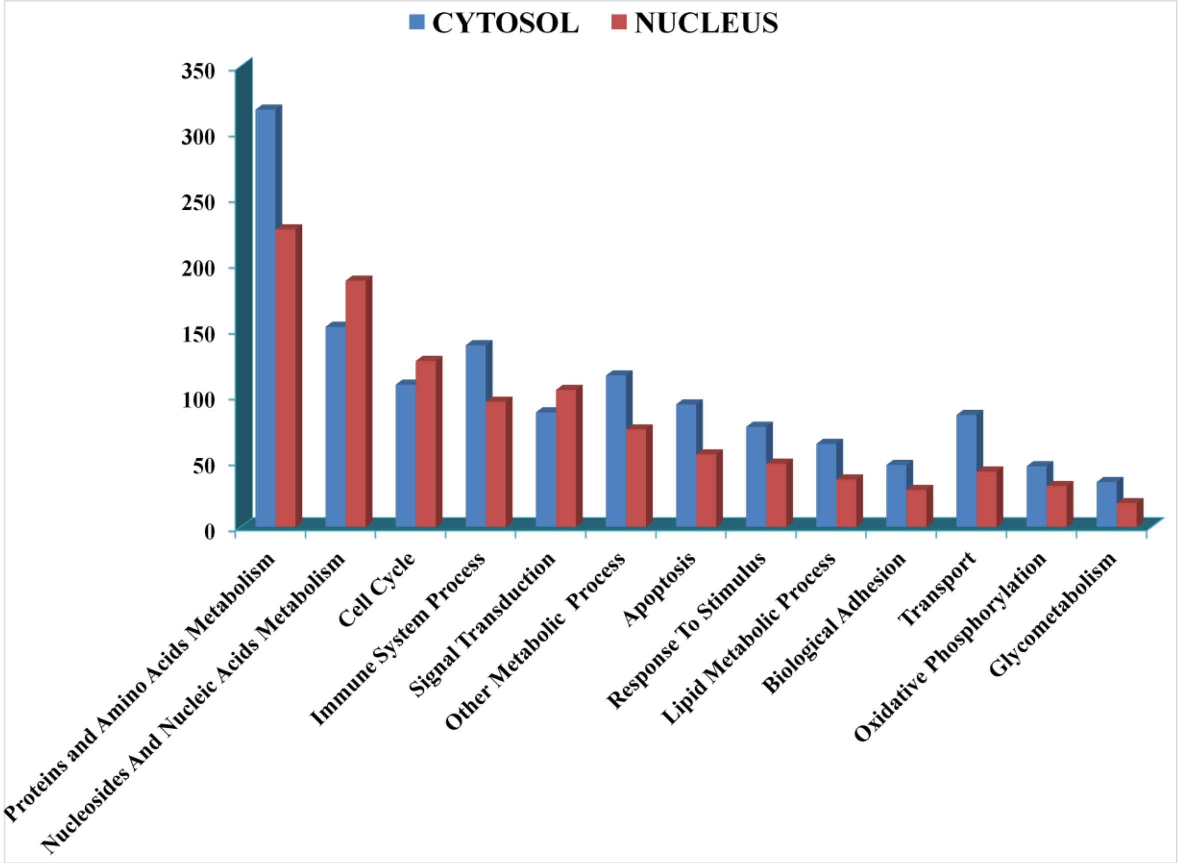

Supplementary Figure S4: Analysis of biological processes and the distribution of differentially expressed proteins identified by MS in PPE26-stimulated macrophages. Proteins whose H/L ratios were greater than 1.2 or less than 0.8 were submitted to PANTHER (http://www.pantherdb.org/) to obtain information about their associated biological processes.

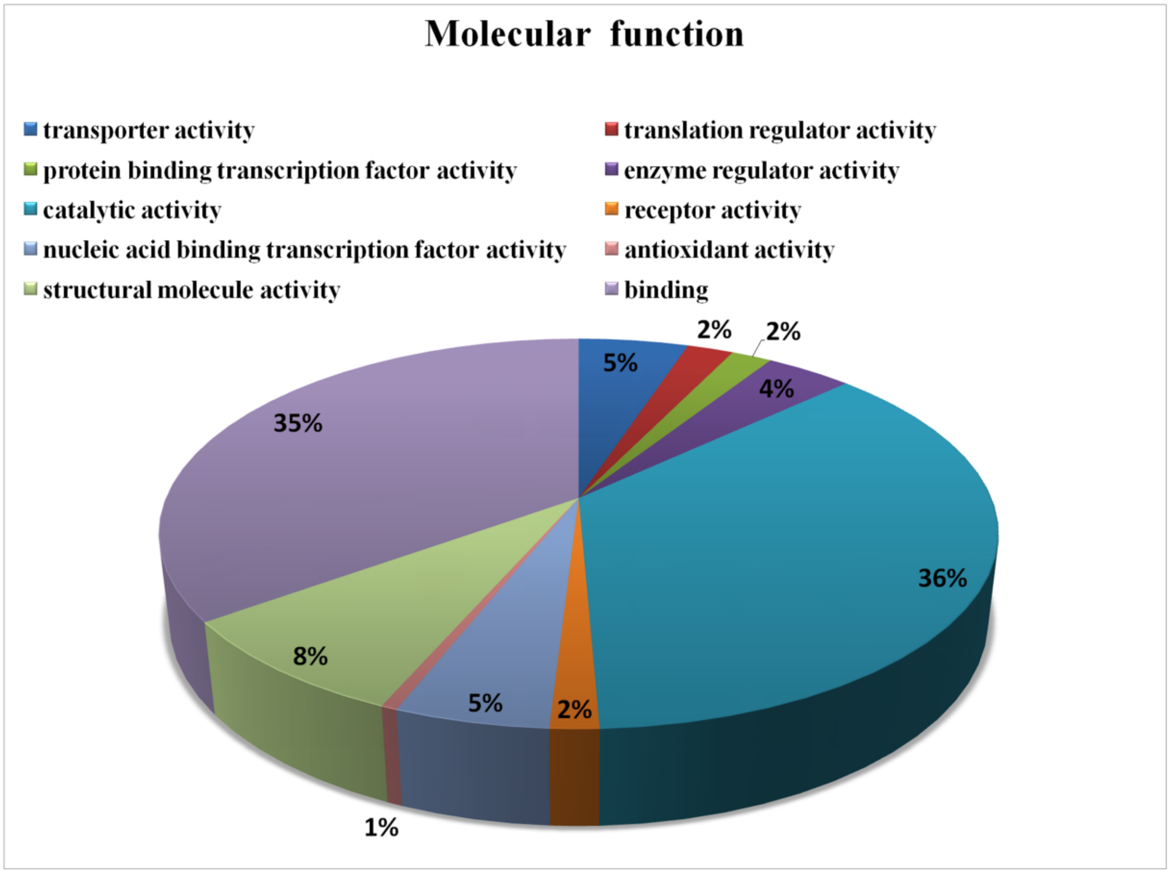

Supplementary Figure S5: Molecular function of the differentially expressed proteins from PPE26-stimulated macrophages identified by MS. Proteins showing an H/L higher than 1.2 or less than 0.8 in the nucleus and cytosol, respectively, were submitted to PANTHER to obtain information about their known functions.

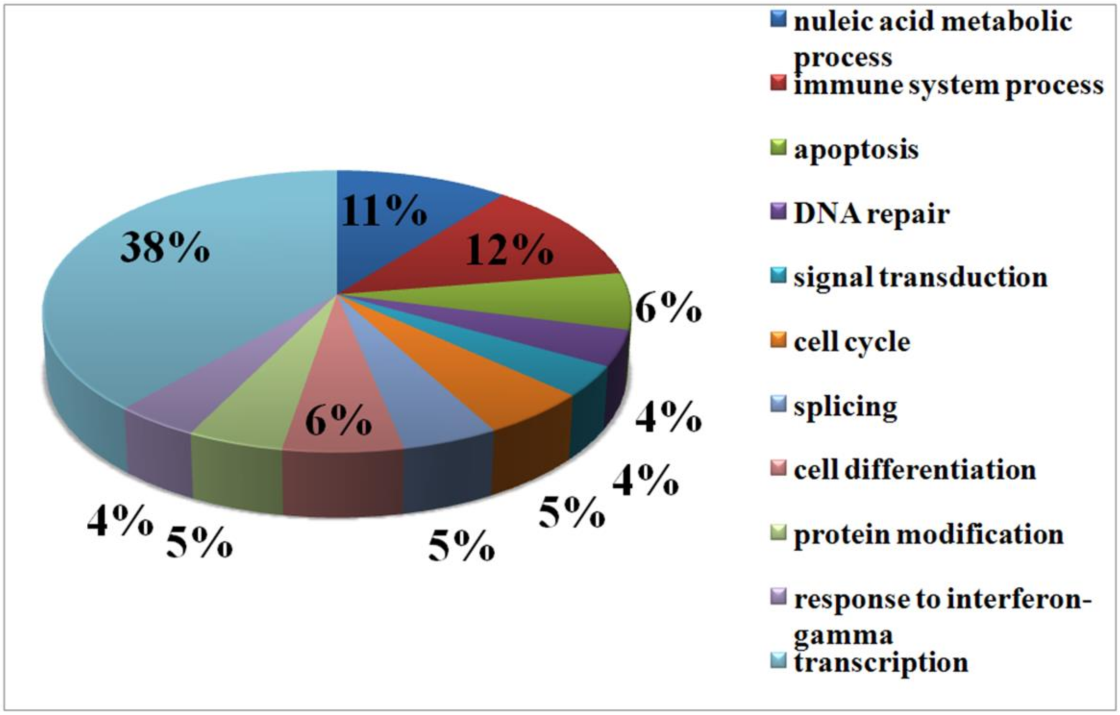

Supplementary Figure S6: Functional analysis of transcriptional factors (TFs). The TFs were submitted to PANTHER to identify related functional processes and corresponding percentages.

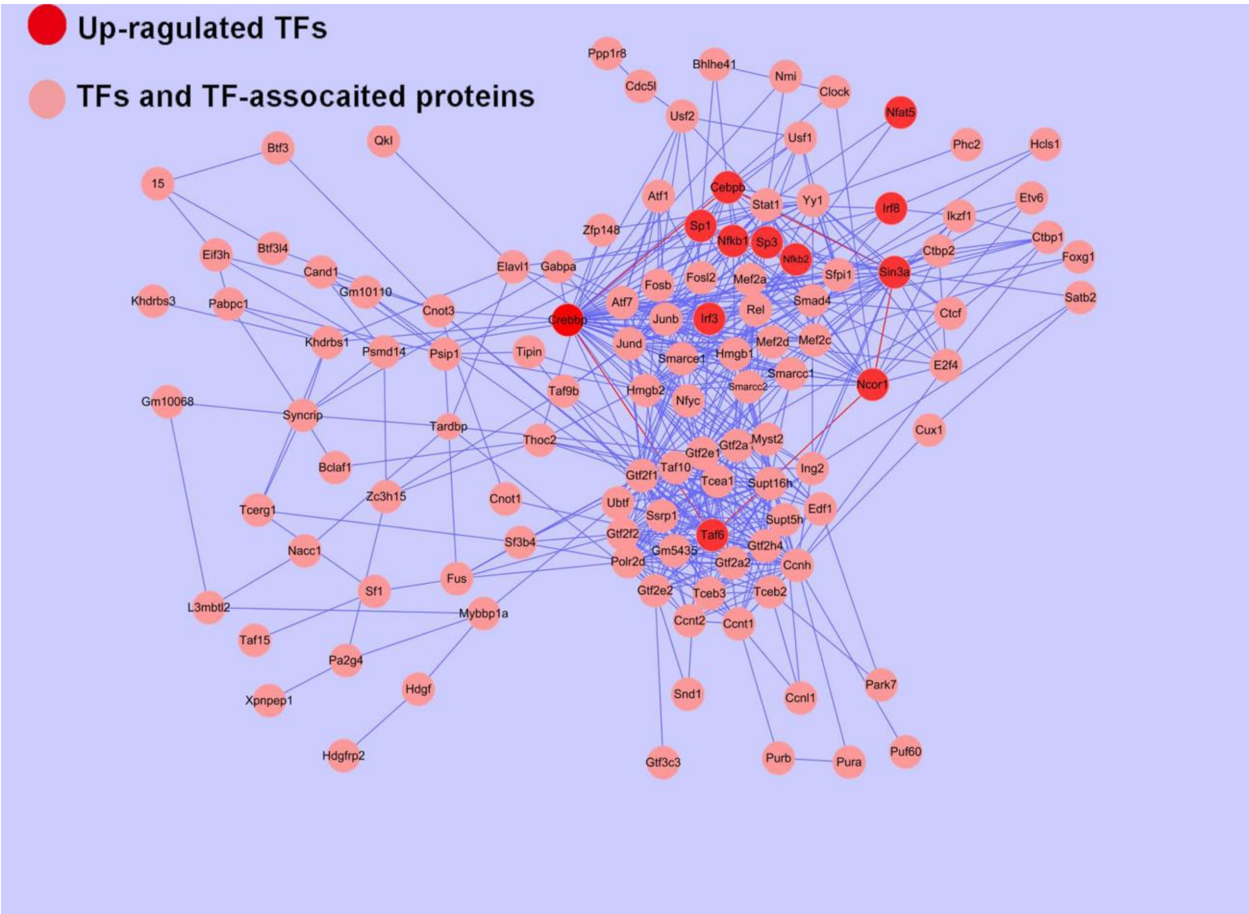

Supplementary Figure S7: The TF-regulatory network operates in conjunction with upstream signal cascades to generate the response following PPE26 stimulation. The TFs and TF-associated factors were submitted to STRING (http://string-db.org/) to reconstruct an interaction network.

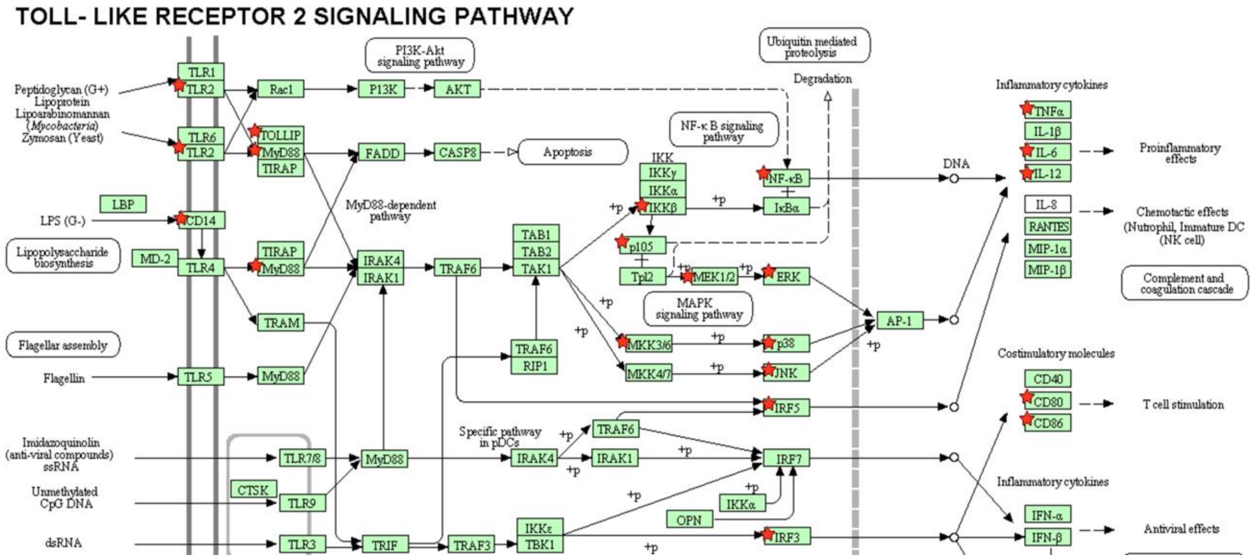

Supplementary Figure S8: Differentially expressed proteins identified in PPE38 stimulated macrophages that are involved in TLR2, MAPKs, NF-κB, IRF Pathway. The differentially expressed proteins identified in PPE38 stimulated macrophages were submitted to DAVID (http://david.abcc.ncifcrf.gov/) to obtain the KEGG pathway regarding antigen processing and presentation. The red stars denote up-regulated proteins.

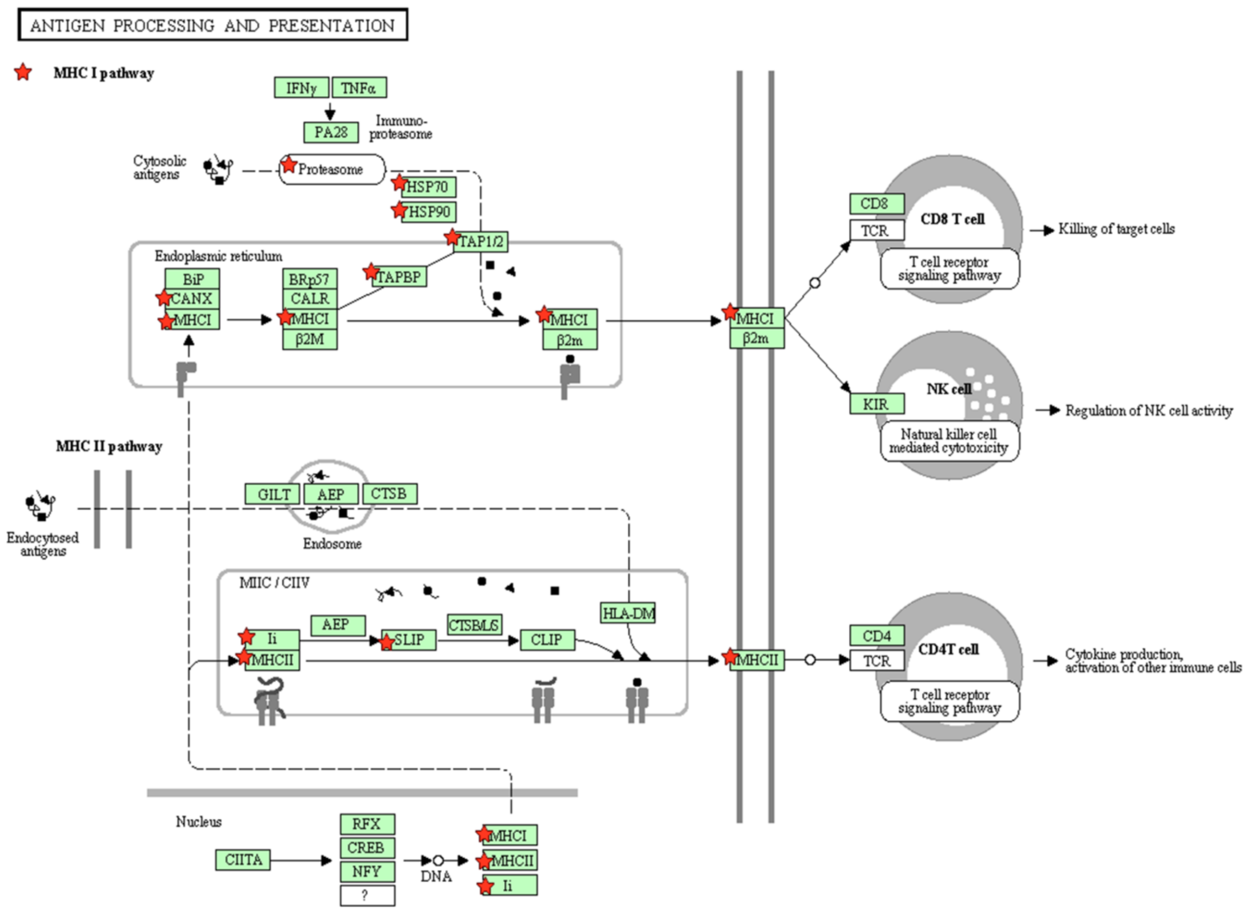

Supplementary Figure S9: Differentially expressed proteins identified in PPE38 stimulated macrophages that are involved in antigen processing and presentation. The differentially expressed proteins identified in PPE38 stimulated macrophages were submitted to DAVID to obtain the KEGG pathway regarding antigen processing and presentation. The red stars denote up-regulated proteins.

Talble 1. Primer Used In The Study		
**Gene ID**	**Primer name**	**Seguence**
recombinant PPE26	forward primer	5′-ATTGAATTCATGGATTTTGGGGCGTTG-3′
recombinant PPE26	reverse primer	5′-ATTGGATCCCTATCCGGCGAAGGGTGG-3′
rBCG-PPE26	forward primer	5′-ATTGAATTCATGGATTTTGGGGCGTTG-3′
rBCG-PPE27	reverse primer	5′-ATTGGATCCCTATCCGGCGAAGGGTGG-3′
b-actin	forward primer	5′-TGCTGTCCCTGTATGCCTCT-3′
b-actin	reverse primer	5′-GGTCTTTACGGATGTCACG- 3′
TNF-a	forward primer	5′-GGCGGTGCCTATGTCTCA-3′
TNF-a	reverse primer	5′-GGCAGCCTTGTCCCTTGA-3′
IL-6	forward primer	5′-TGCCTTCTTGGGACTGAT-3′
IL-6	reverse primer	5′-CTGGCTTTGTCTTTCTTGTT-3′
IL-12p40	forward primer	5′-CAGAAGCTAACCATCTCCTGGTTTG-3′
IL-12p40	reverse primer	5′-TCCGGAGTAATTTGGTGCTTCACAC-3′
